# Medicine in Siam

**Published:** 1845-08

**Authors:** 


*Medicine in Siam.*—Formerly there has been but little demand for Dr. Bradley's labours in the families of any of the nobles (says a missionary letter,) but of late there has been a change which not a little encourages us. Some months since, the head priest of the P'raklang's wat, after suffering a long time with the fever and ague, consented to take quinine, and in a day or two was well. Soon after this, Chau Fa, the priest, having been afflicted in the same way for a long time, and having heard of the above-mentioned cure, consented also to try the quinine; not, however, till he had first proved its virtues in the case of a servant of his. With him, also, the medicine was perfectly successful. Since that time Dr. B. has frequently been called to attend upon the sick in high places. One of the princes of high rank, who but a few months since would not have ventured to take medicine from the foreign doctor, without first requiring one of his servants to take a dose to prove that it contained no poison, now takes it without hesitation directly from the hand of Dr. B., and has even gone so far as to take it while sitting in his boat in front of our dispensary. Some months since, Dr. B. was called to operate for cataract on the eye of a nobleman, who is at the head of the agricultural interests of the kingdom, and equal in rank with the P'raklang. Notwithstanding he is 73 years old, the operation was completely successful. He has evinced his gratitude by a great variety of presents. At one time he sent fifty pails of rice, and a hundred pails of paddy at another. This, at the price rice was bringing at that time, would amount to sixty dollars, and in a time of great scarcity of rice has been of no little service to our hospital.

*Ibid.*

